# Disease-modifying treatments in Alzheimer’s disease

**DOI:** 10.1007/s00415-023-11602-8

**Published:** 2023-02-16

**Authors:** Marc Edwards, Robin Corkill

**Affiliations:** Department of Neurology, University Hospital of Wales, Heath Park, Cardiff, UK

## Introduction

Alzheimer’s disease (AD) remains the leading worldwide cause of dementia. Availability of effective disease-modifying treatments is limited and the only licensed medications in more general use remain symptomatic treatments such as acetylcholinesterase inhibitors and memantine. However, new pharmacological interventions are being explored, based on an understanding of the characteristic pathological processes in AD which are characterised by extracellular deposition of beta-amyloid plaques, development of intracellular neurofibrillary tangles made of phosphorylated tau and progressive neuronal loss. In particular, it is hypothesised that amyloid-targeting therapies may reduce amyloid deposition and hence slow or even stop cognitive decline in AD. In this month’s journal club, we have reviewed randomised clinical trials exploring the use of three of these agents in early AD, two of which (Aducanumab and Lecanemab) have already received approval via the accelerated approval pathway for the treatment of AD from the U.S. Food and Drug Administration (FDA).

## Donanemab in early Alzheimer’s disease

TRAILBLAZER-ALZ was a phase 2, multi-centre, randomised, double-blinded, placebo-controlled trial. Participants were randomised on a 1:1 basis to receive Donanemab every 4 weeks intravenously or placebo. Donanemab is a humanised IgG1 monoclonal antibody which targets established amyloid plaques. Previous trials had demonstrated success in reducing amyloid as measured by positron emission tomography (PET). Participants were aged 60–85 years with an MMSE score of 20–28 and diagnosis of ‘prodromal’ AD [mild cognitive impairment (MCI)] or mild AD dementia.

If amyloid-related imaging abnormalities (ARIA) were seen, Donanemab dose was reduced or switched to placebo. The primary outcome measured the difference in change over 72 weeks using the Integrated Alzheimer’s Disease Rating Scale (IADRS) which is a combination scale derived from the Alzheimer’s Disease Assessment Scale Cognitive Subscale (ADAS-Cog13) and Alzheimer’s Disease Cooperative Study—Activities of Daily Living Inventory (ADCS-iADL), with lower scores indicating greater impairment. Secondary outcome measures included individual components of the IADRS, Clinical Dementia Rating Scale Sum of Boxes (CDR-SB), Mini-Mental State Examination (MMSE) and PET-Amyloid and Tau.

The Donanemab group demonstrated a smaller reduction in IADRS than placebo (− 6.86 vs − 10.06, difference 3.20, *p* = 0.04) and the authors suggest that this represents a lower rate of cognitive and functional decline. However, the majority of the secondary outcomes did not show a statistically significant difference between groups. However, in the Donanemab group, there was a significant reduction of amyloid as seen on PET scan with 67.8% of participants receiving Donanemab switching to placebo by the end of the trial as they achieved ‘amyloid-negative’ status. There was no significant difference in deaths or serious adverse events between the two groups. The main adverse event was amyloid-related imaging abnormality (ARIA) which was statistically more likely in the Donanemab group (26.7% vs 0.8%) although the number of symptomatic participants was lower (6.1% vs 0.8%).

**Comment:** This study demonstrated successful reduction in amyloid burden on PET but it is unclear if this is reflected by clinically significant change. A difference in IADRS score of 6 between treatment and placebo was used in pre-trial calculations to represent a halving of the rate of progression of AD and this was not achieved. The limitations in this study included a lack of ethnic diversity and a significant heterogeneity in Donanemab dosing due to changes made when amyloid levels were reduced on imaging or ARIA observed, which may have also led to unblinding.

*Mintun et al. N Engl J Med 2021; 384:1691–1704*

## Two randomized phase 3 trials of Aducanumab in early Alzheimer’s disease

Efficacy of Aducanumab was assessed via two, almost identical, phase 3, multi-centre, international, placebo-controlled, double-blinded trials studies: EMERGE and ENGAGE. Participants were aged 50–85, diagnosed with MCI due to AD or mild AD and were randomly allocated to receive low-dose Aducanumab, high-dose Aducanumab or placebo in a 1:1:1 ratio. Aducanumab was administered intravenously every 4 weeks over 76 weeks. The dosing in the treatment groups was also stratified depending on ApoE4 carrier status with ApoE4 ε4+ participants receiving a dose of 3 mg/kg (low dose) and 6 mg/kg (high dose). ApoE4 ε4− participants received 6 mg/kg (low dose) and 10 mg/kg (high dose). The minor differences between EMERGE and ENGAGE include start dates and the rates of enrolment.

Two protocol amendments were made during the study and the authors suggest that these may have been the reason for similarly designed trials yielding different results. The first change allowed participants with ARIA to restart the treatment and build to the target dose and second that the ApoE ε4+ high dose group continued up-titration to the maximum 10 mg/kg dose. Due to the enrolment differences 29% of patients in EMERGE achieved full dosing compared with only 22% in ENGAGE.

Criteria for a planned interim futility analysis, designed to terminate the trial early if data suggested the treatment was ineffective, were met. However, the authors argue that assumptions on which the futility analysis was based were violated on two counts. First, that the two studies would yield similar results and second that the treatment effect would not change as the study progressed. The data set for primary efficacy analysis included more data than the dataset used to determine futility and early termination. With the greater volume of data analysed, a statistically significant difference was found in the primary outcome favouring Aducanumab. In the high-dose arm of EMERGE, there was a difference of − 0.39 on the CDR-SB score vs placebo (− 0.69, − 0.09, *p* = 0.012). Three other secondary endpoints also showed statistically significant differences in this arm vs placebo (MMSE, ADAS-Cog13 and ADAS-iADCL-MCI). The ENGAGE study did not yield any statistically significant results between treatment and placebo groups.

**Comment:** The validity of this study has been questioned due to the mid-study protocol changes, early termination and ultimately the significant differences in results of two identically designed trials. Both studies demonstrated successful radiological clearance of amyloid which was dose and time dependent. The low-dose arms in both studies did not yield statistically significant results so that further exploration of high-dose Aducanumab is needed in further studies.

*S. Budd Haeberlein* *,* *et al. J Prev Alz Dis 2022;2(9):197–210*

## Lecanemab in early Alzheimer’s disease

Lecanemab is a human IgG1 monoclonal antibody with a high affinity to soluble amyloid-beta protofibrils. CLARITY-AD was a phase 3 double-blinded and placebo-controlled trial undertaken over 18 months from March 2019 to March 2021. It included centres across North America, Europe and Asia. 1795 participants were randomised on a 1:1 ratio to placebo or Lecanemab intravenous infusion (10 mg/kg) every 2 weeks. Participants were 50–90 years old with a diagnosis of either MCI AD or mild AD dementia using NIAAC criteria with evidence on either amyloid PET imaging or CSF biomarker measurement. The primary outcome compared changes in the CDR-SB which assesses 6 different domains, with a higher score indicating greater impairment. Secondary outcomes included alternate scales assessing severity of AD along with amyloid burden on PET and biomarkers in CSF and plasma.

The Lecanemab group had a statistically significant reduction in worsening as measured by the CDR-SB. The Lecanemab group’s mean score changed by 1.21 points whereas the placebo group changed by 1.66 (difference 0.45, *p* < 0.001). Other cognitive scales assessed (ADCOMS, ADCS-MCI-ADL) also revealed statistically significant changes in favour of Lecanemab. In addition, the Lecanemab arm also had a significant reduction in amyloid seen on PET and all CSF biomarkers improved numerically apart from neurofilament light chain. Serious adverse events were more likely in the Lecanemab group (14% vs 11.3%) which were mainly infusion-related reactions, ARIA-E, atrial fibrillation, syncope and angina.

**Comment:** There are currently ongoing open-label extensions of CLARITY-AD which aim to address one of the main limitations in this study which is its short duration. The COVID-19 pandemic also adversely affected recruitment but was not considered to have affected the outcome. Overall, the results of this study are positive in that Lecanemab was considered to have successfully reduced amyloid deposition and this was matched by statistically significant changes in cognition.

*van Dyck et al. N Engl J Med 2023; 388:9–21*

## Conclusion

 These papers studied three similar therapeutic agents with similar aims. Whilst radiological reduction of amyloid on PET imaging was striking in all studies, the modest reduction in cognitive impairment arguably has limited clinical value at 18 months and may not justify the high costs. It could be argued, however, that the change in trajectory of decline demonstrated at this stage, if maintained, could have significant later clinical benefits to quality and duration of independent life. The rates of ARIA are fairly consistent across the trials and likely to be dose related. A concern with Lecanemab’s safety is the rate of serious cardiovascular adverse events including atrial fibrillation, syncope, and angina. Nevertheless, it has gained FDA approval as of January 2023 and will be welcome news to patients with AD and those close to them. All three agents are involved in ongoing open-label extended studies.

## Data Availability

Not applicable.

